# Evolutionarily Conserved Protein Sequences of Influenza A Viruses, Avian and Human, as Vaccine Targets

**DOI:** 10.1371/journal.pone.0001190

**Published:** 2007-11-21

**Authors:** A. T. Heiny, Olivo Miotto, Kellathur N. Srinivasan, Asif M. Khan, G. L. Zhang, Vladimir Brusic, Tin Wee Tan, J. Thomas August

**Affiliations:** 1 Department of Biochemistry, Yong Loo Lin School of Medicine, National University of Singapore, Singapore, Singapore; 2 Institute of Systems Science, National University of Singapore, Singapore, Singapore; 3 Department of Pharmacology and Molecular Sciences, The Johns Hopkins University School of Medicine, Maryland, United States of America; 4 Product Evaluation and Registration Division, Centre for Drug Administration, Health Sciences Authority, Singapore, Singapore; 5 Department of Microbiology, Yong Loo Lin School of Medicine, National University of Singapore, Singapore, Singapore; 6 Institute for Infocomm Research, Singapore, Singapore; 7 Cancer Vaccine Center, Dana-Farber Cancer Institute, Boston, Massachusetts, United States of America; Utrecht University, Netherlands

## Abstract

**Background:**

Influenza A viruses generate an extreme genetic diversity through point mutation and gene segment exchange, resulting in many new strains that emerge from the animal reservoirs, among which was the recent highly pathogenic H5N1 virus. This genetic diversity also endows these viruses with a dynamic adaptability to their habitats, one result being the rapid selection of genomic variants that resist the immune responses of infected hosts. With the possibility of an influenza A pandemic, a critical need is a vaccine that will recognize and protect against any influenza A pathogen. One feasible approach is a vaccine containing conserved immunogenic protein sequences that represent the genotypic diversity of all current and future avian and human influenza viruses as an alternative to current vaccines that address only the known circulating virus strains.

**Methodology/Principal Findings:**

Methodologies for large-scale analysis of the evolutionary variability of the influenza A virus proteins recorded in public databases were developed and used to elucidate the amino acid sequence diversity and conservation of 36,343 sequences of the 11 viral proteins of the recorded virus isolates of the past 30 years. Technologies were also applied to identify the conserved amino acid sequences from isolates of the past decade, and to evaluate the predicted human lymphocyte antigen (HLA) supertype-restricted class I and II T-cell epitopes of the conserved sequences. Fifty-five (55) sequences of 9 or more amino acids of the polymerases (PB2, PB1, and PA), nucleoprotein (NP), and matrix 1 (M1) proteins were completely conserved in at least 80%, many in 95 to 100%, of the avian and human influenza A virus isolates despite the marked evolutionary variability of the viruses. Almost all (50) of these conserved sequences contained putative supertype HLA class I or class II epitopes as predicted by 4 peptide-HLA binding algorithms. Additionally, data of the Immune Epitope Database (IEDB) include 29 experimentally identified HLA class I and II T-cell epitopes present in 14 of the conserved sequences.

**Conclusions/Significance:**

This study of all reported influenza A virus protein sequences, avian and human, has identified 55 highly conserved sequences, most of which are predicted to have immune relevance as T-cell epitopes. This is a necessary first step in the design and analysis of a polyepitope, pan-influenza A vaccine. In addition to the application described herein, these technologies can be applied to other pathogens and to other therapeutic modalities designed to attack DNA, RNA, or protein sequences critical to pathogen function.

## Introduction

One of the most important threats to human health is infection by avian influenza A viruses [1]–[3]. While global influenza pandemics have occurred only a few times in the past century, the H1N1 pandemic of 1918–1919 caused 20–50 million deaths and was one of the most serious disease outbreaks in recorded history. The recent evolution of the highly lethal avian H5N1 virus, while not transmissible in humans, has emphasized the continued threat of influenza viruses on a global scale. It is widely predicted, given the increased human population and density, that a new pandemic on the scale of the H1N1 infection would have a devastating effect world-wide.

The two currently approved vaccines against influenza viruses are designed specifically to mimic the most recently recognized circulating forms listed in the 2006–2007 influenza prevention and control recommendations (http://www.cdc.gov/mmwr/preview/mmwrhtml/rr5510a1.htm). Both vaccines contain three recently isolated human strains and are subject to possible annual revision of their virus composition. The rapid mutation of the viral HA and NA proteins facilitates the selective replication of new virus strains not subject to immunity based on previous vaccination and is a serious obstacle to the effectiveness of these vaccines [4]–[5]. Alternative vaccine strategies that overcome the problem of rapid viral mutation, can be applied to global populations, and provide for easy production are suggested goals [6]–[8].

The design of a vaccine that guarantees antibody-mediated immunity to new influenza viruses is not currently feasible because the structural determinants of B-cell immunity are highly complex and there is no effective means for predicting the antibody epitope structure of target pathogens. Cell-mediated immunity, in contrast, is based upon the binding of short sequences of antigen proteins, termed T-cell epitopes, to specialized cellular proteins, known as human leukocyte antigens (HLAs), class I (HLA I) and class II (HLA II), that facilitate the presentation of the epitopes to T-cells of the immune system [9]–[14]. The chemical and structural determinants of HLA-peptide binding have been defined for a number of HLA alleles [15]–[19]. Of particular relevance for vaccine design are supertype groupings of similar HLA alleles that display overlapping peptide-binding capacities. The supertypes cover a large fraction of the HLA diversity in the human population and antigen epitopes that bind to the supertypes are considered prime candidates for vaccine formulations [20]–[24]. Supertype-binding motifs and quantitative matrices have been incorporated into several computational prediction algorithms and it is now possible to identify, *in silico,* candidate HLA-restricted T-cell epitopes of protein sequences, allowing large-scale analysis of potential vaccine targets [24]–[27]. Moreover, increasing attention is being given to T-cell-based vaccines because they can be designed as genetic formulations to include selected regions of the viral antigens [28]–[31], and have the many other desirable properties associated with DNA vaccines in general. Studies have demonstrated that epitope-specific T cell responses elicited by immunization with DNA or peptide, and adoptive transfer of epitope-specific T cell clones, could mediate protective immunity, in some cases with single CTL epitopes, against various pathogens in murine experimental models [32]–[42]. Additionally, recent studies have shown that immunization of HLA-A2 transgenic mice against single HLA-A2-restricted T-cell epitopes conferred protection against lethal infection with influenza A virus, vaccinia virus, or LCMV [43]–[45]. Human clinical trials with epitope-based DNA vaccines against HIV [46] and malaria [47] were found to be safe and immunogenic for effector T-cell immune responses but in these first generation studies, failed to achieve the desired clinical goals in the vaccination of healthy volunteers.

Cellular immune responses are recognized to play a role in influenza immunity (for reviews see [48]–[51] and the application of T-cell epitopes has been extensively studied as an alternative to vaccines designed for humoral immunity [52]–[62]. Mouse immunization with DNA encoding NP elicited CTL, IFN-γ and IL-2 responses, with cross-strain protection against virus challenge, and evidence from adoptive transfer, indicated that both types of T cells act as effectors in protective immunity [54]–[55]. Similarly, DNA immunization with H1N1 NP or H5N1 NP or M proteins was found to protect mice against lethal challenge [56]–[58]. However, some influenza vaccine formulations were not successful [59]–[62] and there remain multiple issues for the development of a human T-cell epitope-based vaccines, including epitope selection, delivery systems, epitope processing and presentation, and undoubtedly others.

This study was focused on the large-scale analysis of all influenza A virus protein sequence data of the past 30 years that is recorded in public databases. Information entropy and consensus sequence methodologies were combined to identify sequences of 9 amino acids or longer with a history of complete conservation in 80% or more of both avian and human virus strains. These conserved sequences were further analyzed to identify targets for candidate epitope-based T-cell vaccine formulations against all current and possibly future influenza A pathogens of avian or human origin.

## Methods

### Methodology overview

**Figure 1:**
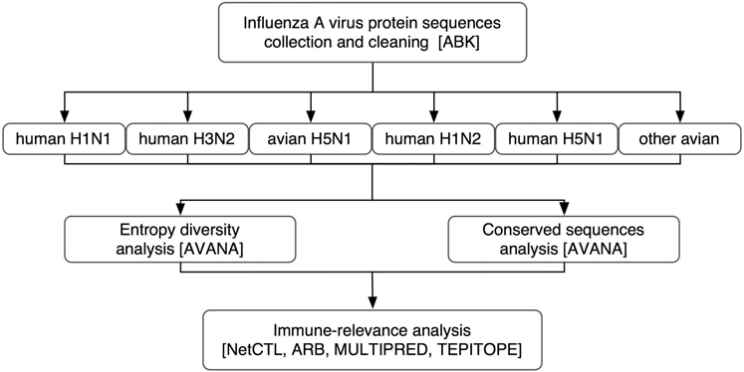
An overview of the methodology of this study.

A general overview of the methodology is depicted in Figure 1. The details and rationale for the systematic approach adopted by this study have been previously published [63]. The primary goal was to identify viral protein sequences that have been conserved over long periods of time, and to select those sequences that have the highest potential for HLA-restricted immunogenicity in a broad spectrum of the human population. The process includes three major steps: i) extraction of all influenza A protein sequence data, processing and definition of data sets comprising relevant human and avian virus groups; ii) entropy analysis of sequence variability and identification of conserved peptide sequences of 9 amino acids or longer; and iii) prediction of supertype-restricted, HLA-binding sequences. Two complementary methods for the identification of conserved sequences were applied: a statistical entropy-based method that takes into account the combinatorial diversity of peptide epitopes and was used to elucidate the variability for different influenza subtypes, and a consensus method, which is robust against sampling biases (such as the predominance of certain influenza subtypes in the dataset), to confirm the conserved sequences. The conserved sequences were then submitted to several epitope prediction programs, whose results are combined. Sequences predicted, and in some cases demonstrated, to contain epitopes to several HLA supertypes are proposed as vaccine epitope candidates because of their wide human population coverage.

### Influenza A virus sequence data collection and processing

A dataset of influenza A protein sequences annotated with isolate name, country and year of isolation, host organism, subtype, and protein name, was derived from all available sequences (as of September 2006) from the NCBI GenBank and GenPept databases, including entries mirrored from the UniProt database. Collection and cleaning of a total of 85,873 records was performed the Aggregator of Biological Knowledge (ABK) [64], which applied structural and semantic rules to automate the aggregation and annotation task. The final set of 36,343 protein sequences was manually verified by two independent curators. Most human influenza subtypes were represented by more than 100 sequences of each viral protein, the count varying depending on the protein. The H1N2 subtype had a lower number of sequences (ranging from 22 to 66) because of its recent emergence [65]. Separate multiple sequence alignments of the 11 proteins were carried out with MUSCLE 3.6 [66]. Because of the great variability exhibited by the HA and NA proteins, separate alignments were obtained for each subtype (16 subtypes for HA and 9 for NA). The subtype alignments were subsequently merged using the MUSCLE tool, to obtain the final HA and NA alignments. The introduction of gaps in the resulting alignments was minimized by merging sequences based on sequence similarity between subtypes, as reported in phylogenetic studies [5], [67]. The in-house developed Antigenic Variability Analyser tool (AVANA) was subsequently used to extract alignments of several subsets of the collected sequences, based on annotation values, such as viral subtype, host, and year of isolation.

### Information entropy analysis

The diversity of the influenza A virus proteome was studied by creating subsets of the influenza A protein sequence alignments, comprising (1) avian sequences, subdivided into 3 decades (1977–1986, 1987–1996, and 1997–2006); (2) H5N1 viruses, subdivided into avian and human isolates; (3) circulating human subtypes, namely H1N1, H3N2, and H1N2. Assuming that each sequence represents an independent isolate, the information entropy methodology [68] was used to measure the variability of influenza A virus proteomes in the context of overlapping nine-amino acid peptides spanning the length of each influenza A protein. The rationale of this selection was the length of peptides that are bound by HLA molecules for presentation to T-cell receptors, typically from 8–20 amino acids, with nine amino acids being the predominant length of class I peptides and the core of class II peptides [69]. Applying Shannon's formula [68], the nonamer peptide entropy *H*(*x)* at any given position *x* in the alignment is computed by
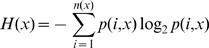
where *p*(*i*,*x*) is the probability of a particular nonamer variant *i* being centered at position *x*. The entropy value increases with *n*(*x*), the total number of variants observed at position *x*; it is also sensitive to the relative frequency of the variants, such that it decreases when one variant is clearly dominant (*i.e.* the position is conserved). Only sequences that contain a valid amino acid at position *x* were used for the entropy computation, and alignment gaps were ignored. Although gaps tend to occur in high-diversity regions, proteins that have a high fraction of gaps have reduced statistical support, yielding an artificially low entropy value; for this reason, positions where more than 50% of sequences contained a gap were discarded. Because of the statistical nature of the entropy measure, both complete protein sequences and shorter fragments were used in this computation.

In theory, nonamer entropy values can range from 0, a completely conserved nonamer sequence in all proteins analyzed, to 2^9^; in practice, however, the upper bound is very much lower for alignments of closely related sequences. For finite-size sets of sequences, entropy computations are affected by the sequence count in the alignment. The effects of alignment size bias are especially noticeable for alignments containing fewer than about 100 sequences, and must be accounted for when making direct comparisons between sequence alignments of different sizes. It has been shown that, for an alignment of *N* sequences, alignment size bias is proportional to 1/*N* [70]. This relationship allows a correction for size bias by applying to each alignment a statistical adjustment that estimates entropy values for an infinitely-sized alignment with analogous variant distribution. To obtain such estimate, the alignment was repeatedly randomly sampled to create smaller alignments of varying size, whose entropy was measured. At each alignment position, the entropy of these subset alignments of size *N* was plotted against 1/*N,* using a linear regression to extrapolate the entropy estimate for *N*→∞. The regression's coefficient of determination (*r* ^2^) was used as a goodness-of-fit of the resulting estimate, confirming the validity of our method (*r* ^2^>0.9 in most cases). In this study, size bias correction was applied to all entropy calculations, so that alignment sequence counts could be ignored in comparisons. All entropy values reported are therefore infinite-size set estimates, rather than the values directly computed from the alignments.

### Conserved influenza A virus sequences

Collected and cleaned influenza A virus records were grouped based on (a) subtype: the circulating human subtypes (H1N1, H3N2, H1N2), H5N1, and other subtypes in avian reservoir; (b) host: human and avian; and (c) year of isolation. The method gave equal weight to all groups and obviated the problem of particular groups being over-represented (such as human H3N2). Six subgroups were derived: (1) human H1N1, (2) human H3N2, (3) human H1N2, (4) human H5N1, (5) avian H5N1, and (6) other avian subtypes. The eleven influenza A proteins of each subgroups were individually aligned using MUSCLE 3.6 [66]. The AVANA tool was used to select nonamers with conservation of ≥80% in each alignment. The minimum length of a conserved sequence was nine amino acids and conserved contiguous nonamers were joined as a single sequence. A consensus sequence (the most frequent sequence) for each conserved sequence in the alignments was generated for each of the 6 subgroups. Corresponding consensus sequences of the subgroups were then aligned and those sequences that were identical in each of the six subgroups and present in at least 80% of all recorded viruses were selected as the highly conserved sequences.

### HLA supertype-restricted T-cell epitopes

The *in silico* prediction of HLA supertype-restricted HLA class I and class II T-cell epitope sequences in the conserved region*s* was performed through four computational systems: NetCTL MULTIPRED, ARB, and TEPITOPE. The NetCTL 1.2 algorithm [25] (http://www.cbs.dtu.dk/services/NetCTL/) predicts peptides restricted to 12 HLA class I supertypes (A1, A2, A3, A24, A26, B7, B8, B27, B39, B44, B58 and B62), integrated with predictions of HLA binding, proteasomal C-terminal cleavage and transport efficiency by the transporter associated with antigen processing (TAP) molecules. HLA binding and proteasomal cleavage predictions are performed by an artificial neural networks (ANN) method and TAP transport efficiency is predicted using a weight matrix method. The parameters used for NetCTL prediction were: 0.15 weight on C terminal cleavage (default), 0.05 weight on TAP transport efficiency (default), and 0.5 threshold for HLA supertype binding.

The Average Relative Binding (ARB) matrix binding prediction method (http://epitope.liai.org:8080/matrix/matrix_prediction.jsp) [27] is allele specific and estimates a matrix of coefficients based upon the association of each of the 20 amino acids at each possible position along the peptide sequence. In this study the data were selected for representative alleles within studied supertypes and predictions are shown for 8 HLA class I alleles of the supertypes A1 (A*0101), A2 (A*0201), A3 (A*0301), A24 (A*2402), A26 (A*2601), B7 (B*0702), and B44 (B*4402, B*4403).

MULTIPRED (http://research.i2r.a-star.edu.sg/multipred/) [26] predicts peptides that bind to HLA class I supertypes A2 (A*0201, *0202, *0203, *0204, *0205, *0206, *0207 and *0209) and A3 (A*0301, *0302, *1101, *1102, *3101, *3301 and *6801)and class II HLA-DR supertype (DRB1*0101, *0401, *1501, *0701, *0901, *1302 and DRB5*0101). Hidden Markov model (HMM) and ANN methods are the predictive engines with sum thresholds of: A2, 31.33 (ANN; SN = 0.80 and SP = 0.83) and 47.08 (HMM; SN = 0.80 and SP = 0.78); A3, 24.53 (ANN; SN = 0.90 and SP = 0.95) and 37.58 (HMM; SN = 0.80 and SP = 0.87); and DR, 23.42 (ANN; SN = 0.90 and SP = 0.92) and 51.08 (HMM; SN = 0.90 and SP = 1.00). TEPITOPE predicts 25 HLA class II (DR) alleles are HLA allele-specific; however, sequences predicted to bind to ≥5 alleles were considered supertypic.

The TEPITOPE software [24] (2000 beta version; obtained by the courtesy of J. Hammer) utilizes quantitative matrix-based motifs, obtained from experimental scanning of the binding of P1-anchored designer peptides to soluble HLA-DR molecules in *in-vitro* competition assays, to predict peptides binding to 25 common HLA-DR alleles (DRB1*0101, *0102, *0301, *0401, *0402, *0404, *0405, *0410, *0421, *0701, *0801, *0802, *0804, *0806, *1101, *1104, *1106, *1107, *1305, *1307, *1311, *1321, *1501, *1502 and DRB5*0101). The parameters for TEPITOPE predictions were: 5% quantitative threshold and putative determinants with a 10-fold inhibitory residue excluded. Predictions were performed for all 25 HLA-DR alleles and nonamer core peptides predicted to bind >5 HLA-DR alleles were selected as supertype-restricted.

### Experimentally identified influenza A T-cell epitopes

T-cell epitope sequences within the conserved sequences were identified by matching the highly conserved sequences and the curated influenza epitope sequences obtained from the Immune Epitope Database and Analysis Resource (www.immuneepitope.org/) [71], [72]. These epitope sequences data were derived from reported HLA binding assays (IC50≤500 nM) or T-cell assays that included ^51^Cr release, HLA tetramer staining, and ELISPOT assays. Only epitope data from unique sequences and containing HLA restriction information were included.

## Results

### Avian and human influenza A virus isolates

The collected and cleaned influenza A virus protein sequences were catalogued in two groups. The recently circulating (1997–2006) influenza A viruses, both avian and human comprising 25,812 sequences of the 11 influenza proteins, both full- and partial-length, from human H1N1 (2,466 sequences), H3N2 (12,199), H1N2 (405), H5N1 (1,055), avian H5N1 (4,361), and all other avian subtypes except H5N1 (5,326) (Table 1). There were over 100 sequences of each protein of every virus with exceptions for the most recent human viruses, H1N2 and H5N1, and the PB1-F2 protein. The second group comprised an additional 10,531 sequences of human H1N1 and H3N2 isolated prior to 1997, and all avian viruses isolated from 1977 to 1986, and 1987 to 1996 (Table 2). The H1N2 sequences before 1997 were excluded because the number of sequences that were available for analysis was insufficient.

**Table 1:** Influenza types A virus protein sequences from the past decade (1997–2006).

Protein	Human H1N1	Human H3N2	Human H1N2	Human H5N1	Avian H5N1^a^	Other Avian^b^	Total
PB2	189	970	33	97	404	401	2,094
PB1	202	984	32	101	400	399	2,118
PB1-F2	183	955	22	47	10	74	1,291
PA	190	970	29	102	402	390	2,083
HA	517	2,032	66	106	657	976	4,354
NP	191	1,012	39	114	420	518	2,294
NA	230	1,245	49	112	577	570	2,783
M1	192	1,024	40	105	458	617	2,436
M2	192	1,045	31	95	289	335	1,987
NS1	190	984	36	95	456	662	2,423
NS2	190	978	28	81	288	384	1,949
Total	2,466	12,199	405	1,055	4,361	5,326	25,812

a All available sequences in the database, mainly from the past decade (1997–2006).

b Other avian subtypes except H5N1, from 1997 to 2006.

**Table 2:** Influenza A virus protein sequences from virus isolates before 1997.

Protein	Human H1N1	Human H3N2	Other Avian^a^		Total
			1977–1986	1987–1996	
PB2	98	337	200	133	768
PB1	106	342	200	134	782
PB1-F2	81	301	181	95	658
PA	96	334	190	135	755
HA	266	1,071	326	252	1,915
NP	133	544	220	153	1,050
NA	142	443	242	145	972
M1	122	398	264	163	947
M2	106	389	258	130	883
NS1	123	373	240	198	934
NS2	111	361	222	173	867
Total	1,384	4,893	2,543	1,711	10,531

a Other avian subtypes of influenza A viruses except H5N1.

### Diversity of influenza A virus proteins

**Figure 2:**
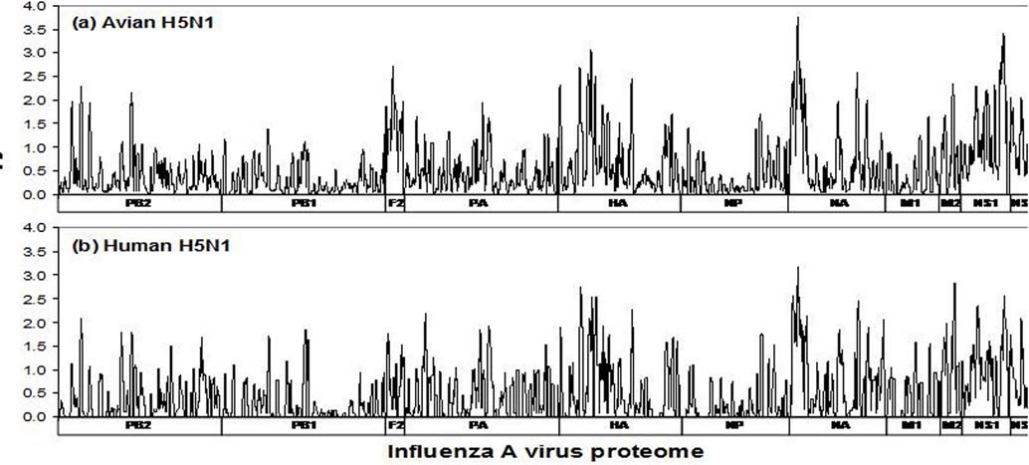
Entropy plots of avian influenza A viruses, excluding H5N1 subtype, for each of three decades: 1977–1986, 1987–1996, 1997–2006 (data as of September 30, 2006).

The diversity in the protein sequences of influenza A viruses was examined by application of the information entropy methodology to each 9 amino acid sequence of the viral proteins. Data of the past 30 years comprised 9,640 avian influenza A subtype sequences (1977–1986, 2,543 sequences; 1987–1996, 1,711; 1997–2006, 5,326 (Table 1 and 2, Figure 2). The gross patterns of protein variability of the avian viruses from each of the past 3 decades were very similar in the context of the relative diversity of the proteins. The viral surface glycoproteins, HA and NA, showed extreme sequence diversity, illustrative of the reassortment of the genome segments among the many subtypes of the avian group A viruses as well as the rapid rate of point mutation, with multiple amino acids at virtually every position (entropy >2.0) except at a single region in HA that has remained remarkably conserved despite the extreme sequence modification of every other nonamer of the protein. The PB1-F2, NS1 and NS2, and to a lesser extent M2, also showed a history of high variability. In contrast, the polymerase proteins (PB2, PB1, and PA), as well as the NP and M1, contained many historically highly conserved regions (entropy <1.0). The overall gradual increase in entropy over the three decades in many of the protein sequences, most apparent in the highly conserved sequences, is an indication of the continuing genetic evolution of the viruses as well as improved screening of sequence variants. However, these changes do not distort the overall pattern of highly conserved and highly variable sequences.

**Figure 3 pone-0001190-g003:**
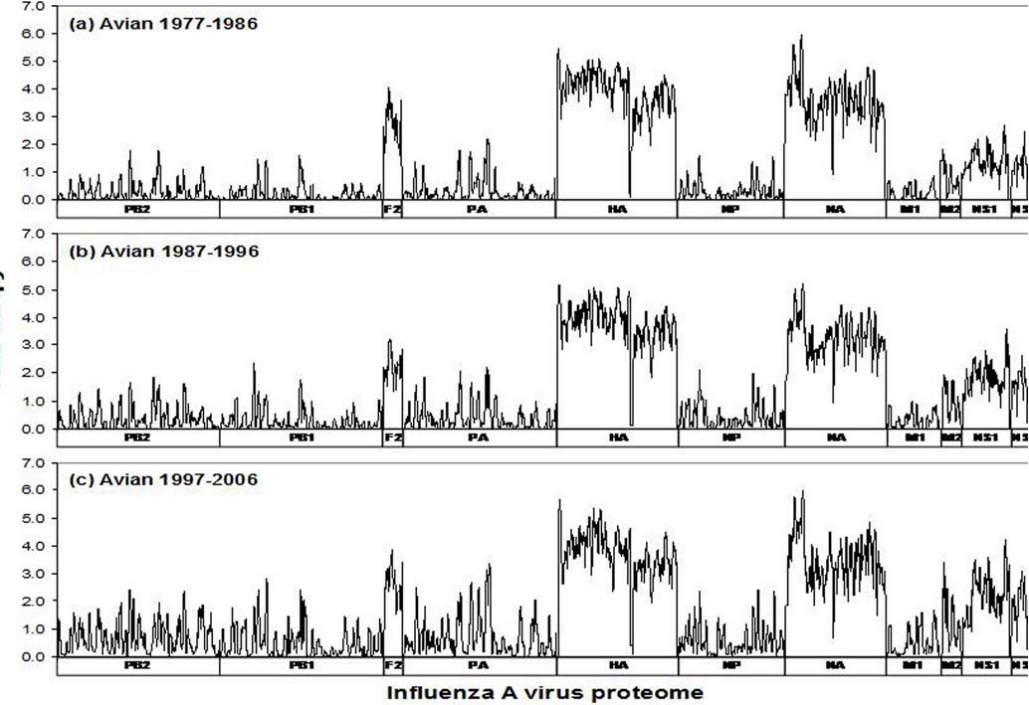
Entropy plots of the sequence alignments of recorded H5N1 viruses isolated from avian and human hosts (data as of September 30, 2006).

H5N1 protein entropy patterns of the 1997 to 2006 isolates from humans (1,055 sequences) and birds (4,361) were grossly similar and reflect the high mutational variability in amino acid composition of proteins of even a single subtype influenza A virus (Figure 3). Of the eleven known influenza A proteins, only the short PB1-F2 protein, the product of an alternative ORF of the PB1 RNA segment [73] showed notable differences when comparing the diversity profiles of the two groups. There is evidence that PB1-F2 is involved in the apoptosis of host immune cells, increased viral virulence in a mouse model, and destruction of alveolar macrophages [74], and the limited diversity of this protein in human isolates may have relevance to H5N1 virulence and pathogenicity. In the context of the remaining proteins, the similarity of human and avian H5N1 entropy patterns is consistent with the observations that, to date, all human H5N1 isolates represent avian to human transmission from isolated clusters of avian infection. Moreover, detailed analyses of mutations associated with human-to-human transmission have shown that all human H5N1 virus isolates have a predominant avian footprint [75]

**Figure 4:**
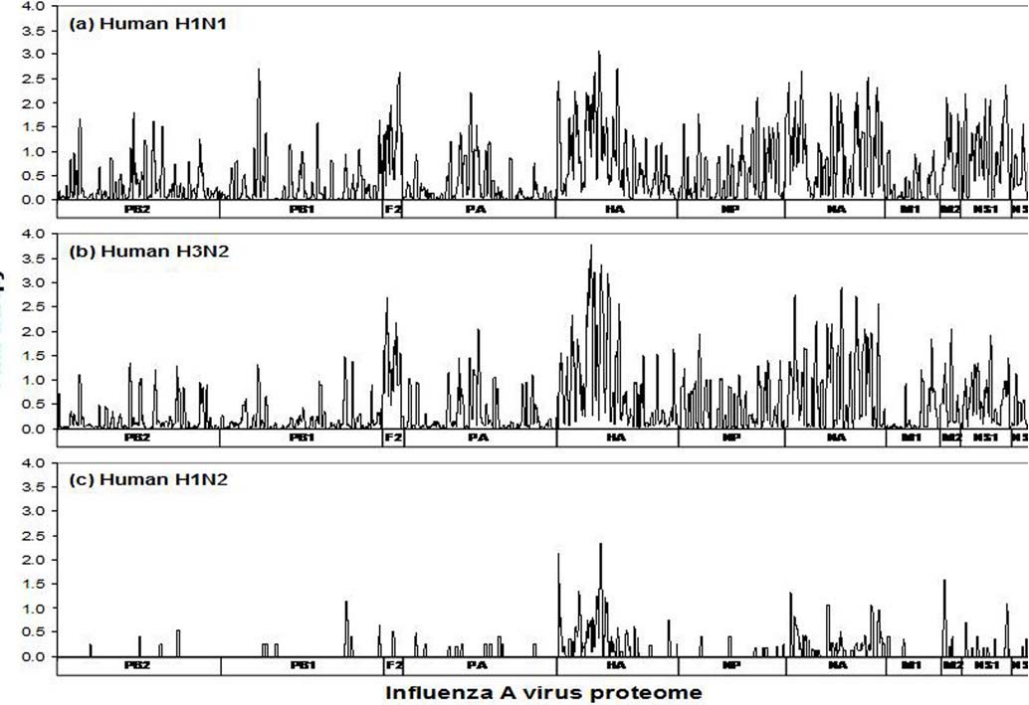
Entropy plots of recorded human influenza A subtypes H1N1, H3N2, and H1N2 from 1918–2006 (data as of September 30, 2006).

Entropy of the protein sequences of each of the three circulating human viruses isolated between 1918 and 2006 (H1N1, 3,850 sequences; H3N2, 17,092; and H1N2, 414 [including 9 sequences before 1997]) reflect different patterns of sequence evolution (Figure 4). The complex protein sequence diversity pattern of the human H1N1 reflects its mutational evolution from its avian characteristics at the time of human transmission in 1918 to a sequence characteristic of human H1N1, with the greatest evolutionary diversity in the HA, NA, PB1-F2, NS1 and NS2, and to a lesser extent M2, similar to the diversity of the viruses in the avian host. There subsequently was further evolution of the human subtypes by gene segment exchange, resulting in H2N2 in 1957, H3N2 in 1968, and H1N2 in 1988. The continuing mutational modification of H1N2 and H3N2 have resulted in entropy patterns distinctive of the human transmitted influenza A viruses with a large number of amino acid sequence patterns that differ from those of the avian to avian counterpart. In contrast, the most recent H1N2 human subtype that appeared in 1988 (www.cdc.gov/flu/about/h1n2.htm) continues to exhibit limited evolutionary variability with many identical or highly conserved sequences regions in all of the few (22 to 66) recorded individual protein sequences (see Table 1). It is likely that the human H1N2 virus evolved from a very limited, perhaps single reassortment of the HA gene segment in the case of an individual infected with both of the human transmitted H1N1 and H3N2 viruses.

**Figure 5:**
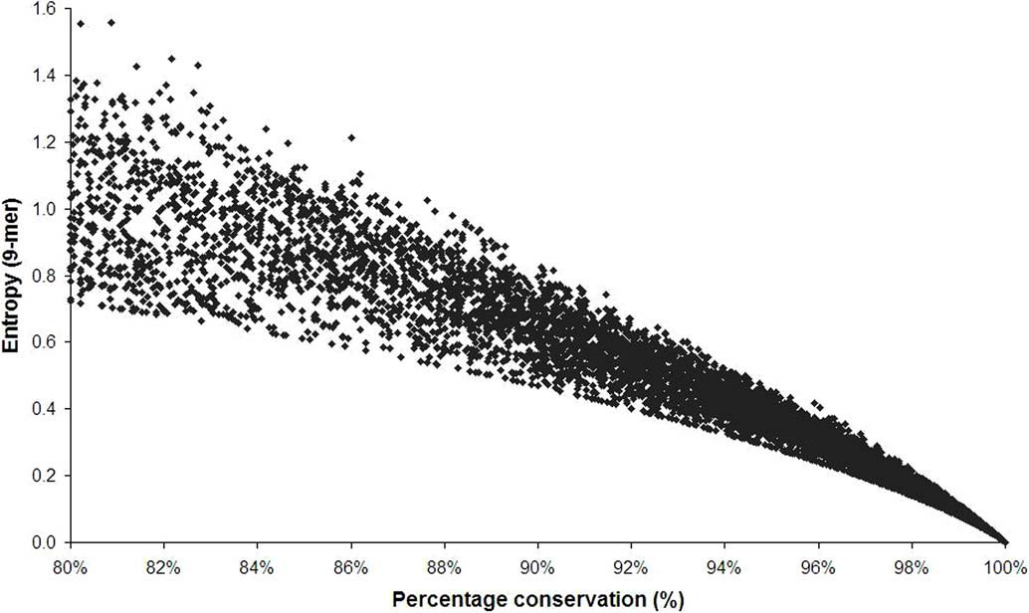
Entropy-sequence conservation relationship, plotted from data in this study (see Figure 2–4). The boxed region indicates area whereby conservation of ≥90% correlates to entropy of 0.8 or less.

The nature of the entropy distribution of the conserved sequences is not demonstrated in these data as entropy is not a linear function but is defined both by the number of sites and frequency of variability. A given entropy value can be related to a high fraction of different amino acids at one site and limited variability at other amino acid sites, or to limited variability at a large number of amino acid sites. This absence of a direct correlation of entropy to the degree of sequence conservation is seen in the markedly diverse nonamer entropy values (∼0.7 to 1.5) of the collected sequences with 80% conservation (Figure 5). A more limited range of entropy values can be associated with sequence conservation of 90–100%.

We concluded that the PB2, PB1, PA, NP, and M1 proteins of all recorded influenza A viruses, both avian and human, contain sequences of low variability and high conservation despite differences in evolutionary pathway, subtypes, and host species. These sequences with a history and predicted future of low variability are prime targets for epitope-based T-cell vaccine formulations.

### Amino acid composition of the highly conserved sequences

**Figure 6:**
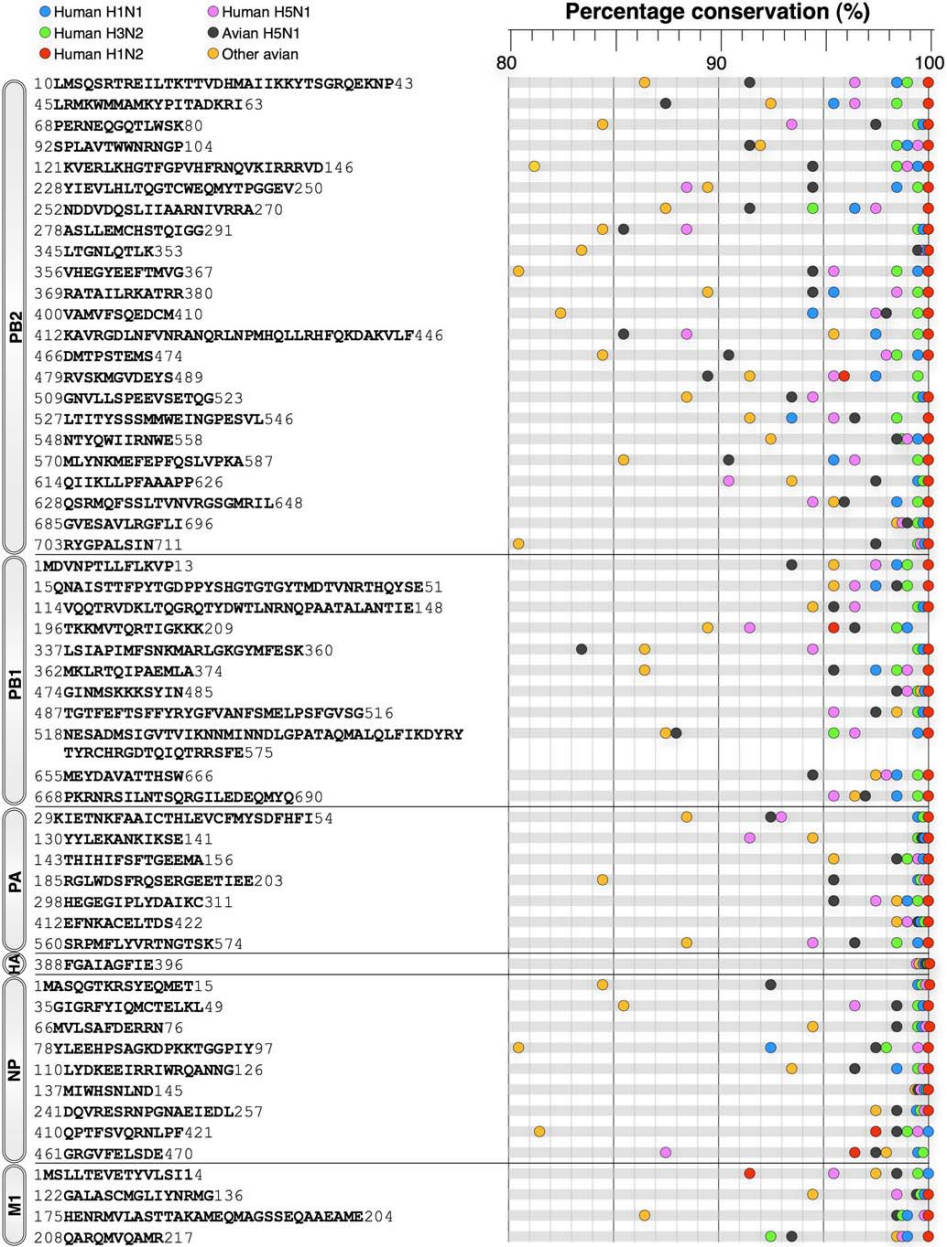
Highly conserved sequences of influenza A viruses in human H1N1, H3N2, H1N2, H5N1, avian H5N1, and other avian subtypes circulating between 1997 and 2006. A region in the viral proteome is considered as highly conserved when it has identical sequence conservation of at least 9 contiguous amino acids in 80% or more of the protein sequences of the analyzed dataset. The index of virus colored symbol is as shown at the top of the figure.

A total of 55 peptide sequences, ranging from 9 to 58 amino acids in length, and containing a total of 965 amino acids, ∼21% of the total proteome (Table 3), were completely conserved in 80% to100% of the human and avian type A viruses recorded in the past decade (Figure 6, Table S1). Twenty-six (26) were present in 90% to100% of the viruses. The majority of the conserved sequences were in the nonstructural (NS) proteins. PB2 was the most conserved with 23 sequences, comprising 50% of the protein, conserved in 80% to 100% of the documented viruses (Table 3). PB1 was also highly conserved (11 sequences, 36%) and the PA, NP, and M1 proteins contained significant fractions (16% to 27%) of conserved sequences. HA contained one sequence, FGAIAGFIE, that was conserved in all type A viruses despite the extreme variability of all other HA amino acids (see Figure 2). There were no sequences in the PB1-F2, NA, M2, NS1 or NS2 proteins that were completely conserved in at least 80% of the viruses.

**Table 3:** The influenza A virus proteins, their length, the number of conserved sequences, and the combined length of the conserved sequences of each protein

Protein	Length (aa)^a^	Number of highly conserved sequences^b^	Total length of conserved sequences (aa)^c^
PB2	759	23	379 (50%)
PB1	757	11	271 (36%)
PB1-F2	90	0	0
PA	716	7	111 (16%)
HA	568	1	9 (2%)
NP	498	9	126 (25%)
NA	469	0	0
M1	252	4	69 (27%)
M2	97	0	0
NS1	230	0	0
NS2	121	0	0
Total	4,557	55	965 (21%)^d^

a Based on the complete genome sequences of A/Goose/Guangdong/1/96 (H5N1), Taxonomy ID: 93838.

b Number of high conserved sequences with sequence and nonamer conservation of ≥80% in influenza A virus sequences from 1997 to 2006 (human H1N1, human H3N2, human H1N2, human H5N1, avian H5N1, and other avian subtypes) in each of the 11 proteins.

c The sum of highly conserved sequences length in each of the 11 proteins. The numbers in parentheses indicate the percentage of highly conserved sequences length over the total protein length.

d The percentage of total highly conserved sequences length over total influenza A proteome length.

The H1N1, H3N2, and H1N2 viruses circulating in humans had the highest representation of conserved sequences, with almost all of the 55 sequences present in 95% to 100% of the isolates of each virus. All but one (22 of 23) of the H1N2 PB2 conserved sequences were identical in each of the virus isolates. By comparison, only 62% to 76% of the conserved sequences of the avian and human H5N1 subgroups, respectively, and only 33% of the conserved sequences of all other avian subtypes were found in 95–100% of the isolates. The greater proportion of conserved sequences in the human isolates can be attributed to the more recent history and limited rate of evolution the influenza viruses transmitted by humans. This is especially true of the human H1N2 virus, the most recent human influenza A virus.

### HLA-restricted T-cell epitopes

**Figure 7:**
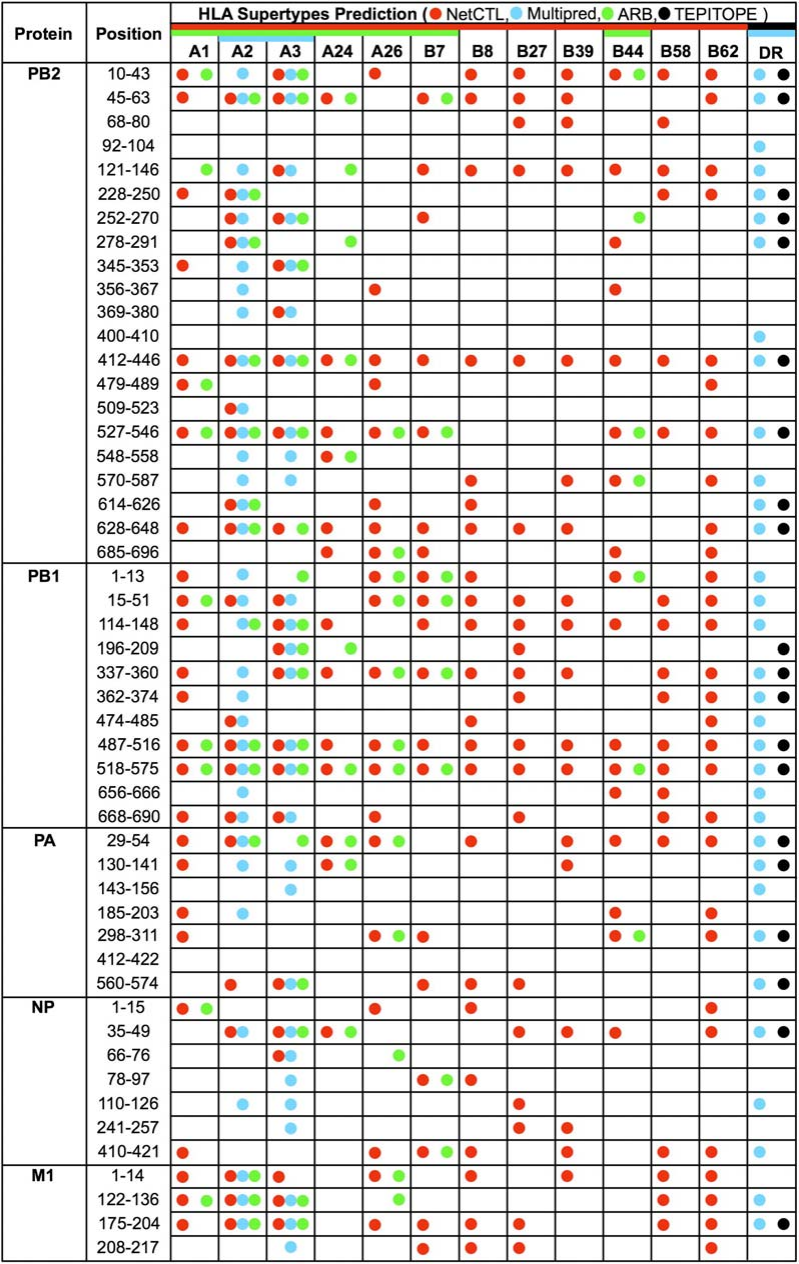
Highly conserved sequences of influenza A viruses and their predicted HLA class I and II supertype-restricted T-cell epitopes by NetCTL, ARB, TEPITOPE, and MULTIPRED systems. The color symbols corresponding to the prediction systems are as shown at the top of the figure. Only conserved sequences containing predicted alleles are shown. NetCTL predicts all of the listed class I supertypes; MULTIPRED predictions cover A2 and A3; and ARB predicts each of the class I except B8, B27, B39, B58, and B62. Predictions of HLA class II supertypes by MULTIPRED AND TEPITOPE is described in Materials and Methods.

The association of conserved sequences and T-cell epitopes was examined by (a) *in silico* prediction of HLA-restricted binding sequences corresponding to supertype alleles by TEPITOPE [24], NetCTL [25], MULTIPRED [26] and ARB [27] algorithms; and (b) reported experimental HLA-binding and T-cell assay data. Most of the peptides representing the conserved sequences (50 of 55) were predicted to contain class I and/or class II binding sequences (Figure 7). There was no significant difference in the density of predicted epitopes in the conserved as compared to non-conserved sequences (data not shown). The detailed listing of nonamer sequences of the conserved regions and the predicted supertypes of these specific nonamers in shown as a supplement (Table S2). For example over 500 HLA class I and over 100 class II HLA binding sequences of supertype alleles were predicted, with many of the nonamer sequences predicted to bind to multiple (2 to 9) individual class I alleles. Similarly, all of the DR binding predictions were selected as supertypes on the basis of predicted binding to multiple DR-alleles (individual predictions not shown). The consistency of class I predictions by the different algorithms ranged from 31% to 66% in those supertypes (A1, A2, A3, A24, A26, B7, B44) where more than one computational system was available. The highest consistency of binding sequences cross-predicted by more than one system was observed with A2 (57%), A3 (66%), and DR (56%).

**Figure 8:**
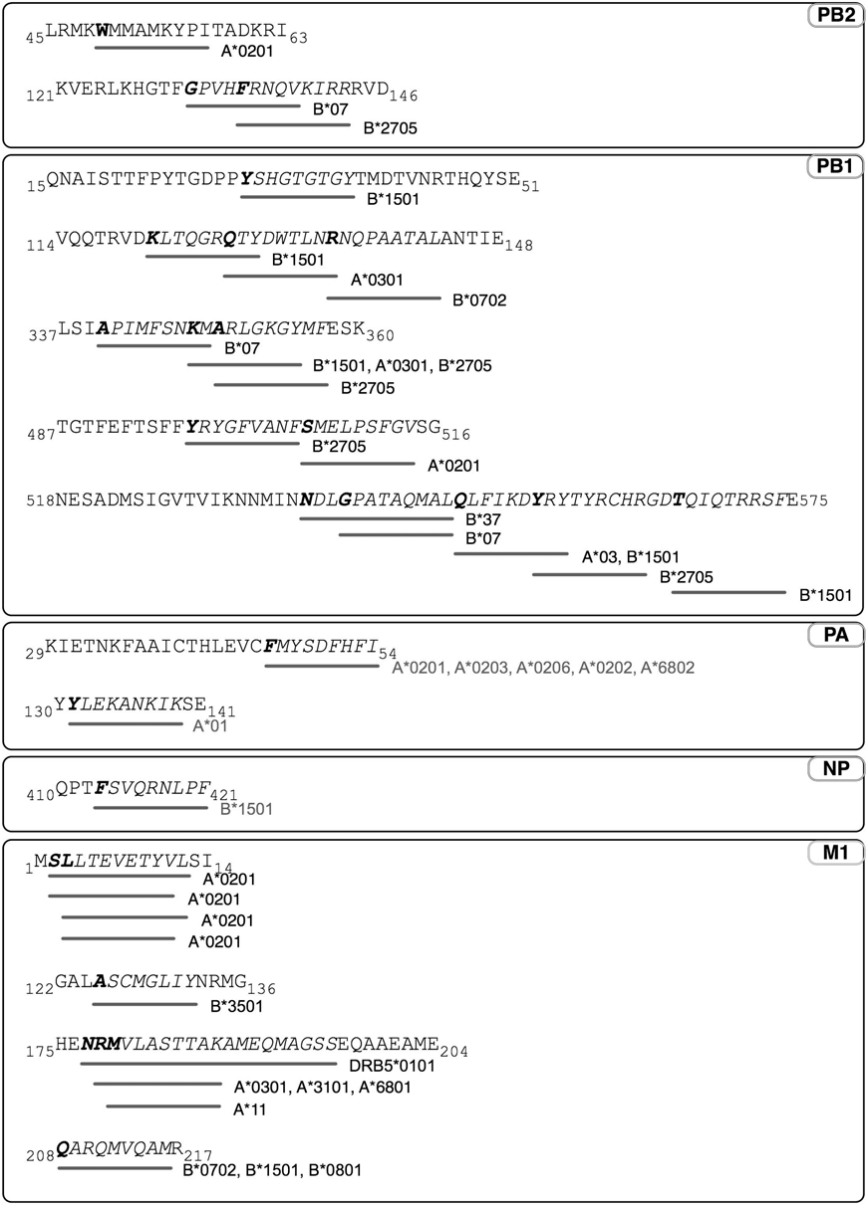
Highly conserved sequences of influenza A viruses and their associated HLA-restricted T-cell epitope based on data obtained from IEDB (www.immuneepitope.org/). Only sequences with identified sites are included. The first amino acid of each identified allele is shown in bold.

Fourteen (14) of the 55 conserved regions contained a total of 29 reported T-cell epitopes based on T-cell assay and/or HLA-binding data entered into the Immune Epitope Database and Analysis Resource (www.immuneepitope.org/) (Figure 8). These 14 experimentally derived sequences included all of the predicted HLA supertypes of the M1 protein, and 5 of the 11 predicted PB1 supertypes. The majority, 22 of the 29 reported T-cell epitopes, were present as clusters (hotspots) of 2 or more overlapping or closely associated reported epitopes; for example, PB1 518-575 contains 5 epitope sequences (9–10 amino acids) between position 537 and 574. Some of the sequences were promiscuous in their association with multiple supertype alleles, for example, the PA 29-54 sequence containing the nonamer FMYSDFHFI that was experimentally shown to bind to at least 5 class I supertype alleles (A*0201, A*0203, A*0206, A*0202, and A*6802).

All but one of these 29 unique influenza A HLA epitopes reported in the IEDB and located in the conserved sequences are class I. This HLA distribution differs markedly from the corresponding total IEDB reported influenza A epitopes representing the complete viral proteome, which show a much greater representation, almost 50%, of class II epitopes: 225 class I and 95 class II. Because the conserved sequences represent ∼21% of the total proteome, if there were a random distribution of T-cell epitopes in the viral proteins, one could expect about 45 class I and 20 class II epitopes in the conserved sequences, as compared to the observed 28:1. These data are consistent with the conventional model that T-cell epitopes derived from the PB2, PB1, PA, NP, and M1 nonstructural proteins that contain the conserved sequences would be processed primarily in the cytoplasmic proteosomal class I pathway.

## Discussion

The marked variability of influenza A virus surface proteins, the major targets of the neutralizing antibodies, have posed a serious obstacle in the development of effective and long-lasting influenza vaccines. As a possible solution, we have identified virus protein sequences that are completely conserved in the majority of all recorded genomic variants that have evolve from avian reservoirs, both avian and human. The information entropy methodology for analysis of protein variability was modified to examine sequences of 9 amino acids or longer, instead of the more common application to single residues, as a means to relate the conserved sequences to the immune function of HLA-restricted peptides. This use of entropy methodology for the identification of highly conserved protein sequences ushers a new experimental strategy in the development of vaccines for pathogens with high rates of mutation. The comprehensive analysis of conserved sequences may also have other applications to pathogen diagnosis or therapy. These sequences are known or can be presumed to have critical roles in viral survival and thus are choice targets for the development of antiviral agents.

Many reports, particularly with respect to the human immunodeficiency virus type 1 (HIV-1) have described a strategic advantage in the use of computational analysis and conserved sequences for vaccine design [76]–[83]. Additionally, the analysis of sequence and immunology databases for the relationship between amino acid sequences and CTL epitope distributions indicate a localization of CTL epitopes in conserved regions of proteins [84]. In contrast, the highly variable regions that lacked epitopes showed evidence of past immune escape with an enrichment of amino acids that do not serve as C-terminal anchor residues and a paucity of predicted proteasome processing sites [85]–[86]. Likewise, the high genetic variability with continually evolving variants of influenza viruses favors sequence modifications at all sites that result in enhanced virus propagation or survival by adaptation to the host cell immune response. Therefore, a vaccine based upon sequences that are naturally highly conserved in all influenza A viruses may greatly restrict the range of possible mutants that could selectively overcome immune suppression. Such a vaccine would have significant strategic advantage provided the sequences have immune function capability, the design of the immunogen is compatible with the requirements for appropriate immune processing and presentation of the protein, and the epitopes have sufficient HLA-representation to cover the global distribution of HLA genotypes. It appears that these requirements can be satisfied given the large number of predicted supertype MHC binding sequences in the conserved regions of the influenza proteins, the experimental reports of T-cell epitopes of the conserved sequences, and our findings of T-cell responses by HLA transgenic mice to almost all conserved sequences of West Nile virus (unpublished data).

A question, however, is why influenza A differs from other pathogens that elicit immune responses to natural infection or vaccination that prevent repeated infection. It is evident that the mechanisms involved in the immune response to influenza A virus infection are in some manner more complex. A discerning report [87] addresses the ecological and immunological determinants of influenza evolution in relation to several of the characteristic features of influenza infection; i.e., the marked replacement of existing strains during a pandemic caused by antigenic shift, the short-lived viral sublineages that characterize influenza A infection and evolution, and the marked seasonality of influenza incidence. A proposed model [86] to address these characteristic features of influenza infection and evolution was that the host immune system responds in a manner that inhibits immediate re-infection but is short-lived with a time scale of weeks to months and is nonspecific to intra- and inter-subtypes. This pattern of short-lived, cross-reactive immunity points to an initial cytotoxic T-lymphocyte (CTL) response that does not persist. We attribute this to the extreme variability of the structural proteins of influenza A viruses, especially that of the HA and NA proteins. Studies of mice and model pathogens suggest that the initial response of naive CD8^+^ T-cells to antigen requires only a brief stimulation with antigen early in the immune response, in a matter of hours, for the cells to become activated, divide, and differentiate into short lived effector cells [88]–[90]. This initial activation can occur in the absence of T-cell help, but without the CD4^+^ response, the quality of the cytotoxic response to antigen challenge after priming gradually decreases and fails to respond effectively to secondary encounters with antigen. Data of several studies indicate that generation of long term CD8^+^ T-cell immune memory requires the concurrent function of professional antigen presenting cells for class II antigen processing and presentation to CD4^+^ helper T-cells during the initial antigen priming period [91]–[93]. It is likely that the major sources of T-cell epitopes, both class I and II, early after influenza infection are those proteins delivered to the immune system by the virus, including the highly variable structural proteins, HA and NA. Thus, this initial response, and the memory T-cells elicited by this response, may lack the highly conserved epitope sequences of the non-structural proteins that would be synthesized at a later stage of infection and, as cytoplasmic proteins, function primarily as endogenous class I epitopes. In this context, it is noteworthy that of the 29 reported influenza T-cell epitopes found in conserved sequences, there was only a single class II epitope, further suggesting that following natural infection, the conserved sequences elicit primarily cytotoxic T-cell responses.

We suggest that a vaccine composed of conserved influenza A virus sequences may provide a memory immunity to non-structural proteins of all viral variants as a means for augmenting the natural response to the virus structural proteins and to provide an enhanced and augmented immune response to any newly emerging avian influenza A pathogen, as well as to the persistence of mutant forms of human transmitted influenza A. This study establishes the identity of all the highly conserved sequences of both human and avian influenza proteomes as the first step in the selection of these sequences for the synthesis of a supertype, epitope-based genetic vaccine.

## Supporting Information

Table S1 Highly conserved sequences of influenza A viruses and their occurrence in each subgroup. ^a^ Highly conserved sequences refer to sequences with ⩾80% conservation in each of the six groups that were analyzed. ^b^ The percentage conservation (rounded down as whole numbers) was calculated as the number of sequences that are identical to the highly conserved sequences divided by the total number of sequences in the same position. The numbers in square brackets indicate the total number of unique sequences at the considered position, inclusive of the highly conserved sequences. ^c^ The total number of human H1N1 sequences ranged from 187 to 242. ^d^ The total number of human H3N2 sequences ranged from 969 to 1141. ^e^ The total number of human H1N2 sequences ranged from 24 to 40. ^f^ The total number of human H5N1 sequences ranged from 82 to 106. ^g^ The total number of avian H5N1 sequences ranged from 217 to 648. ^h ^The total number of avian influenza A subtypes sequences ranged from 210 to 633.
(0.26 MB DOC)

Table S2 Potential HLA-restricted binding sequences in the highly conserved sequences of influenza A virus that are predicted by the NetCTL, ARB, TEPITOPE, and MULTIPRED systems. ^a^ Highly conserved sequences of influenza A viruses (Figure 4) and nonameric binding sequences predicted by NetCTL, ARB, TEPITOPE, and/or MULTIPRED algorithms. The numbers in parentheses indicate the number of nonameric binding sequences in a highly conserved sequence that was predicted by at least one algorithm. ^b^ Nonamers that bind to HLA class I were predicted using NetCTL, ARB, and MULTIPRED. NetCTL 1.2 Server predicts for T cell epitopes that bind to 12 MHC I supertypes, by integrating MHC binding, proteasomal C terminal cleavage, and TAP transport efficiency. MULTIPRED predicts for potential HLA supertype-restricted nonameric sequences that bind to two HLA class I (A2 and A3) supertypes. Only sequences that were predicted by both artificial neural network (ANN) and hidden markov model (HMM) are included. ARB predicts for T-cell epitopes that bind to 30 MHC class I alleles and 12 class II alleles. This study focused on class I alleles that are the most common in each supertype (according to Lund et al., 2004), namely class I A*0101 in A1 supertype, A*0201 in A2 supertype, A*0301 in A3 supertype, A*2402 in A24 supertype, A*2601 in A26 supertype, B*0702 in B7 supertype, B*4402 and B*4403 in B44 supertype. Only sequences, 9aa for class I that were predicted to bind to these common alleles are listed. Nonamers that were predicted to bind in any one of the three systems are listed. ^c^ Nonamers that bind to HLA class II were predicted using TEPITOPE and MULTIPRED. TEPITOPE predicts for T cell epitopes that bind to 25 MHC II alleles. Only promiscuous nonameric sequences that were predicted to bind to at least 5 alleles by TEPITOPE system were listed and indicated as “DR”. MULTIPRED predicts for potential HLA supertype-restricted nonameric sequences that bind to 8 HLA DRB1 alleles. Only sequences that were predicted by both artificial neural network (ANN) and hidden markov model (HMM) are included. Nonamers that were predicted to bind in any one of the two systems are listed.
(0.55 MB DOC)
