# Supplementary material for: Evolutionarily Conserved Protein Sequences of Influenza A Viruses, Avian and Human, as Vaccine Targets
Source: PLoS One. 2007 Nov 21;2(11):e1190. doi: 10.1371/journal.pone.0001190 (PMC2065905; doi:10.1371/journal.pone.0001190)
Supplement: Table S1 — Highly conserved sequences of influenza A viruses and their occurrence in each subgroup. a Highly conserved sequences refer to sequences with ⩾80% conservation in each of the six groups that were analyzed. b The percentage conservation (rounded down as whole numbers) was calculated as the number of sequences that are identical to the highly conserved sequences divided by the total number of sequences in the same position. The numbers in square brackets indicate the total number of unique sequences at the considered position, inclusive of the highly conserved sequences. c The total number of human H1N1 sequences ranged from 187 to 242. d The total number of human H3N2 sequences ranged from 969 to 1141. e The total number of human H1N2 sequences ranged from 24 to 40. f The total number of human H5N1 sequences ranged from 82 to 106. g The total number of avian H5N1 sequences ranged from 217 to 648. h The total number of avian influenza A subtypes sequences ranged from 210 to 633. (0.26 MB DOC) [file pone.0001190.s001.doc]

| **Protein** | Highly conserved sequences^a^ | **Sequence Conservation and Number of Unique Sequences^b^** | | | | | |
| --- | --- | --- | --- | --- | --- | --- | --- |
|  |  | **Human** | **Human** | **Human** | **Human** | **Avian** | **Other** |
|  |  | **H1N1^c^** | **H3N2^d^** | **H1N2^e^** | **H5N1^f^** | **H5N1^g^** | **Avian^h^** |
| **PB2** | 10-LMSQSRTREILTKTTVDHMAIIKKYTSGRQEKNP-43 (34) | 98% [4] | 98% [11] | 100% [1] | 96% [4] | 91% [15] | 86% [9] |
|  | 45-LRMKWMMAMKYPITADKRI-63 (19) | 95% [4] | 98% [9] | 100% [1] | 96% [3] | 87% [8] | 93% [14] |
|  | 68-PERNEQGQTLWSK-80 (13) | 99% [2] | 99% [5] | 100% [1] | 93% [5] | 97% [8] | 84% [14] |
|  | 92-SPLAVTWWNRNGP-104 (13) | 98% [3] | 98% [4] | 100% [1] | 100% [1] | 92% [8] | 92% [7] |
|  | 121-KVERLKHGTFGPVHFRNQVKIRRRVD-146 (26) | 100% [1] | 98% [11] | 100% [1] | 98% [2] | 94% [11] | 81% [28] |
|  | 228-YIEVLHLTQGTCWEQMYTPGGEV-250 (23) | 98% [3] | 99% [4] | 100% [1] | 88% [4] | 94% [9] | 89% [15] |
|  | 252-NDDVDQSLIIAARNIVRRA-270 (19) | 96% [2] | 94% [4] | 100% [1] | 97% [2] | 91% [10] | 87% [9] |
|  | 278-ASLLEMCHSTQIGG-291 (14) | 100% [1] | 99% [4] | 100% [1] | 88% [3] | 85% [6] | 84% [10] |
|  | 345-LTGNLQTLK-353 (9) | 100% [1] | 99% [2] | 100% [1] | 100% [1] | 99% [3] | 83% [5] |
|  | 356-VHEGYEEFTMVG-367 (12) | 99% [2] | 98% [8] | 100% [1] | 95% [4] | 94% [8] | 80% [11] |
|  | 369-RATAILRKATRR-380 (12) | 95% [4] | 99% [4] | 100% [1] | 98% [2] | 94% [8] | 89% [9] |
|  | 400-VAMVFSQEDCM-410 (11) | 94% [2] | 99% [3] | 100% [1] | 97% [2] | 97% [5] | 82% [7] |
|  | 412-KAVRGDLNFVNRANQRLNPMHQLLRHFQKDAKVLF-446 (35) | 98% [4] | 99% [6] | 100% [1] | 88% [3] | 85% [11] | 95% [10] |
|  | 466-DMTPSTEMS-474 (9) | 99% [2] | 98% [11] | 100% [1] | 97% [3] | 90% [11] | 84% [15] |
|  | 479-RVSKMGVDEYS-489 (11) | 97% [3] | 99% [6] | 95% [2] | 95% [4] | 89% [9] | 91% [10] |
|  | 509-GNVLLSPEEVSETQG-523 (15) | 100% [1] | 99% [2] | 100% [1] | 94% [2] | 93% [9] | 88% [5] |
|  | 527-LTITYSSSMMWEINGPESVL-546 (20) | 93% [2] | 98% [5] | 100% [1] | 95% [2] | 96% [6] | 91% [8] |
|  | 548-NTYQWIIRNWE-558 (11) | 100% [1] | 98% [4] | 100% [1] | 98% [2] | 98% [6] | 92% [4] |
|  | 570-MLYNKMEFEPFQSLVPKA-587 (18) | 95% [4] | 99% [5] | 100% [1] | 96% [3] | 90% [6] | 85% [18] |
|  | 614-QIIKLLPFAAAPP-626 (13) | 99% [2] | 99% [3] | 100% [1] | 90% [3] | 97% [4] | 93% [11] |
|  | 628-QSRMQFSSLTVNVRGSGMRIL-648 (21) | 98% [3] | 99% [5] | 100% [1] | 94% [5] | 95% [12] | 95% [9] |
|  | 685-GVESAVLRGFLI-696 (12) | 100% [1] | 99% [3] | 100% [1] | 98% [2] | 98% [4] | 98% [5] |
|  | 703-RYGPALSIN-711 (9) | 100% [1] | 99% [4] | 100% [1] | 100% [1] | 97% [3] | 80% [7] |
| **PB1** | 1-MDVNPTLLFLKVP-13 (13) | 98% [4] | 98% [8] | 100% [1] | 97% [2] | 93% [10] | 95% [7] |
|  | 15-QNAISTTFPYTGDPPYSHGTGTGYTMDTVNRTHQYSE-51 (37) | 97% [5] | 98% [8] | 100% [1] | 96% [4] | 98% [5] | 95% [16] |
|  | 114-VQQTRVDKLTQGRQTYDWTLNRNQPAATALANTIE-148 (35) | 99% [2] | 99% [5] | 100% [1] | 96% [3] | 95% [10] | 94% [11] |
|  | 196-TKKMVTQRTIGKKK-209 (14) | 98% [3] | 98% [3] | 95% [2] | 91% [5] | 96% [11] | 89% [20] |
|  | 337-LSIAPIMFSNKMARLGKGYMFESK-360 (24) | 99% [2] | 99% [8] | 100% [1] | 94% [3] | 83% [7] | 86% [15] |
|  | 362-MKLRTQIPAEMLA-374 (13) | 97% [5] | 98% [10] | 100% [1] | 98% [2] | 95% [13] | 86% [14] |
|  | 474-GINMSKKKSYIN-485 (12) | 100% [1] | 99% [4] | 100% [1] | 98% [2] | 98% [6] | 99% [2] |
|  | 487-TGTFEFTSFFYRYGFVANFSMELPSFGVSG-516 (30) | 100% [1] | 99% [4] | 100% [1] | 95% [4] | 97% [4] | 98% [4] |
|  | 518-NESADMSIGVTVIKNNMINNDLGPATAQMALQLFIKDYRYTYRCHRGDTQIQ | 99% [2] | 95% [15] | 100% [1] | 96% [3] | 87% [17] | 87% [16] |
|  | TRRSFE-575 (58) |  |  |  |  |  |  |
|  | 655-MEYDAVATTHSW-666 (12) | 98% [2] | 99% [2] | 100% [1] | 97% [3] | 94% [6] | 97% [3] |
|  | 668-PKRNRSILNTSQRGILEDEQMYQ-690 (23) | 98% [3] | 99% [3] | 100% [1] | 95% [3] | 96% [8] | 96% [7] |
| **PA** | 29-KIETNKFAAICTHLEVCFMYSDFHFI-54 (26) | 99% [2] | 99% [5] | 100% [1] | 92% [4] | 92% [13] | 88% [19] |
|  | 130-YYLEKANKIKSE-141 (12) | 100% [1] | 99% [5] | 100% [1] | 91% [2] | 99% [2] | 94% [5] |
|  | 143-THIHIFSFTGEEMA-156 (14) | 100% [1] | 98% [6] | 100% [1] | 100% [1] | 98% [4] | 95% [11] |
|  | 185-RGLWDSFRQSERGEETIEE-203 (19) | 99% [2] | 99% [3] | 100% [1] | 100% [1] | 95% [10] | 84% [17] |
|  | 298-HEGEGIPLYDAIKC-311 (14) | 98% [3] | 99% [3] | 100% [1] | 97% [2] | 95% [9] | 98% [5] |
|  | 412-EFNKACELTDS-422 (11) | 100% [1] | 100% [1] | 100% [1] | 98% [2] | 99% [3] | 98% [5] |
|  | 560-SRPMFLYVRTNGTSK-574 (15) | 99% [2] | 98% [7] | 100% [1] | 94% [3] | 96% [8] | 93% [8] |
| **HA** | 388-FGAIAGFIE-396 (9) | 99% [2] | 99% [4] | 100% [1] | 99% [2] | 99% [3] | 99% [5] |
| **NP** | 1-MASQGTKRSYEQMET-15 (15) | 99% [2] | 99% [4] | 100% [1] | 100% [1] | 92% [11] | 84% [9] |
|  | 35-GIGRFYIQMCTELKL-49 (15) | 99% [2] | 99% [4] | 100% [1] | 96% [3] | 98% [6] | 85% [8] |
|  | 66-MVLSAFDERRN-76 (11) | 100% [1] | 99% [2] | 100% [1] | 100% [1] | 98% [3] | 94% [7] |
|  | 78-YLEEHPSAGKDPKKTGGPIY-97 (20) | 92% [2] | 97% [6] | 100% [1] | 100% [1] | 97% [7] | 80% [12] |
|  | 110-LYDKEEIRRIWRQANNG-126 (17) | 98% [3] | 99% [3] | 100% [1] | 100% [1] | 96% [6] | 93% [13] |
|  | 137-MIWHSNLND-145 (9) | 100% [1] | 100% [1] | 100% [1] | 100% [1] | 99% [3] | 99% [2] |
|  | 241-DQVRESRNPGNAEIEDL-257 (17) | 99% [2] | 99% [3] | 100% [1] | 100% [1] | 98% [8] | 97% [7] |
|  | 410-QPTFSVQRNLPF-421 (12) | 100% [1] | 98% [4] | 97% [2] | 99% [2] | 98% [3] | 81% [7] |
|  | 461-GRGVFELSDE-470 (10) | 99% [2] | 99% [4] | 96% [2] | 92% [2] | 97% [5] | 97% [5] |
| **M1** | 1-MSLLTEVETYVLSI-14 (14) | 100% [1] | 99% [3] | 91% [2] | 95% [3] | 98% [4] | 97% [7] |
|  | 122-GALASCMGLIYNRMG-136 (15) | 100% [1] | 99% [2] | 100% [1] | 98% [2] | 99% [4] | 94% [8] |
|  | 175-HENRMVLASTTAKAMEQMAGSSEQAAEAME-204 (30) | 98% [3] | 98% [8] | 100% [1] | 100% [1] | 98% [6] | 86% [21] |
|  | 208-QARQMVQAMR-217 (10) | 98% [3] | 92% [7] | 100% [1] | 98% [2] | 93% [4] | 98% [4] |
