# Supplementary material for: Evolutionarily Conserved Protein Sequences of Influenza A Viruses, Avian and Human, as Vaccine Targets
Source: PLoS One. 2007 Nov 21;2(11):e1190. doi: 10.1371/journal.pone.0001190 (PMC2065905; doi:10.1371/journal.pone.0001190)
Supplement: Table S2 — Potential HLA-restricted binding sequences in the highly conserved sequences of influenza A virus that are predicted by the NetCTL, ARB, TEPITOPE, and MULTIPRED systems. a Highly conserved sequences of influenza A viruses (Figure 4) and nonameric binding sequences predicted by NetCTL, ARB, TEPITOPE, and/or MULTIPRED algorithms. The numbers in parentheses indicate the number of nonameric binding sequences in a highly conserved sequence that was predicted by at least one algorithm. b Nonamers that bind to HLA class I were predicted using NetCTL, ARB, and MULTIPRED. NetCTL 1.2 Server predicts for T cell epitopes that bind to 12 MHC I supertypes, by integrating MHC binding, proteasomal C terminal cleavage, and TAP transport efficiency. MULTIPRED predicts for potential HLA supertype-restricted nonameric sequences that bind to two HLA class I (A2 and A3) supertypes. Only sequences that were predicted by both artificial neural network (ANN) and hidden markov model (HMM) are included. ARB predicts for T-cell epitopes that bind to 30 MHC class I alleles and 12 class II alleles. This study focused on class I alleles that are the most common in each supertype (according to Lund et al., 2004), namely class I A*0101 in A1 supertype, A*0201 in A2 supertype, A*0301 in A3 supertype, A*2402 in A24 supertype, A*2601 in A26 supertype, B*0702 in B7 supertype, B*4402 and B*4403 in B44 supertype. Only sequences, 9aa for class I that were predicted to bind to these common alleles are listed. Nonamers that were predicted to bind in any one of the three systems are listed. c Nonamers that bind to HLA class II were predicted using TEPITOPE and MULTIPRED. TEPITOPE predicts for T cell epitopes that bind to 25 MHC II alleles. Only promiscuous nonameric sequences that were predicted to bind to at least 5 alleles by TEPITOPE system were listed and indicated as “DR”. MULTIPRED predicts for potential HLA supertype-restricted nonameric sequences that bind to 8 HLA DRB1 alleles. Only sequences that w [file pone.0001190.s002.doc]

| **Protein** | **Highly conserved sequences^a^** | **Class I^b^** | **Class II^c^** |
| --- | --- | --- | --- |
|
| **PB2** | 10-LMSQSRTREILTKTTVDHMAIIKKYTSGRQEKNP-43 (17) |  |  |
|  | LMSQSRTRE | − | DR |
|  | MSQSRTREI | B8 | − |
|  | SQSRTREIL | B39, B44, B62 | − |
|  | SRTREILTK | A3, B27 | − |
|  | REILTKTTV | A2, B44 | − |
|  | ILTKTTVDH | A2 | DR |
|  | LTKTTVDHM | A26 | DR |
|  | KTTVDHMAI | B58 | − |
|  | TTVDHMAII | A2, A26 | − |
|  | TVDHMAIIK | A1, A3 | − |
|  | VDHMAIIKK | A3 | − |
|  | DHMAIIKKY | A1, A26 | − |
|  | HMAIIKKYT | A2 | − |
|  | MAIIKKYTS | − | DR |
|  | IIKKYTSGR | A3 | DR |
|  | IKKYTSGRQ | − | DR |
|  | KYTSGRQEK | A3 | − |
|  | 45-LRMKWMMAMKYPITADKRI-63 (9) |  |  |
|  | LRMKWMMAM | B8, B27, B39 | DR |
|  | RMKWMMAMK | A3, B8, B27 | − |
|  | MKWMMAMKY | A1, A3, B27, B62 | DR |
|  | WMMAMKYPI | A2, A24, B7, B8, B27, B62 | DR |
|  | MMAMKYPIT | A2 | DR |
|  | MAMKYPITA | A2, A3 | DR |
|  | MKYPITADK | A3, B27 | − |
|  | KYPITADKR | A24 | − |
|  | YPITADKRI | B7 | − |
|  | 68-PERNEQGQTLWSK-80 (2) |  |  |
|  | ERNEQGQTL | B27, B39 | − |
|  | RNEQGQTLW | B58 | − |
|  | 92-SPLAVTWWNRNGP-104 (1) |  |  |
|  | LAVTWWNRN | − | DR |
|  | 121-KVERLKHGTFGPVHFRNQVKIRRRVD-146 (11) |  |  |
|  | VERLKHGTF | A24, B8, B44, B62 | DR |
|  | LKHGTFGPV | − | DR |
|  | HGTFGPVHF | B58 | − |
|  | GTFGPVHFR | A1, A3 | − |
|  | GPVHFRNQV | B7 | − |
|  | PVHFRNQVK | A3 | − |
|  | VHFRNQVKI | A2, B39 | DR |
|  | HFRNQVKIR | A3 | − |
|  | FRNQVKIRR | A3, B27 | DR |
|  | RNQVKIRRR | A3 | − |
|  | QVKIRRRVD | B8 | − |
|  | 228-YIEVLHLTQGTCWEQMYTPGGEV-250 (8) |  |  |
|  | YIEVLHLTQ | A1 | − |
|  | VLHLTQGTC | − | DR |
|  | LHLTQGTCW | B58 | − |
|  | LTQGTCWEQ | − | − |
|  | QGTCWEQMY | A1 | − |
|  | GTCWEQMYT | B62 | − |
|  | WEQMYTPGG | − | DR |
|  | QMYTPGGEV | A1, A2, B62 | − |
|  | 252-NDDVDQSLIIAARNIVRRA-270 (7) |  |  |
|  | DVDQSLIIA | A2 | − |
|  | VDQSLIAA | B44 | − |
|  | SLIIAARNI | A2 | − |
|  | LIIAARNIV | A2 | DR |
|  | IIAARNIVR | A3 | DR |
|  | IAARNIVRR | A2, A3 | DR |
|  | AARNIVRRA | B7 | − |
|  | 278-ASLLEMCHSTQIGG-291 (3) |  |  |
|  | SLLEMCHST | A2 | − |
|  | LEMCHSTQI | A24, B44 | DR |
|  | MCHSTQIGG | − | DR |
|  | 345-LTGNLQTLK-353 (1) |  |  |
|  | LTGNLQTLK | A1, A3 | − |
|  | 356-VHEGYEEFTMVG-367 (2) |  |  |
|  | HEGYEEFTM | B44 | − |
|  | EGYEEFTMV | A2, A26 | − |
|  | 369-RATAILRKATRR-380 (2) |  |  |
|  | TAILRKATR | A3 | − |
|  | AILRKATRR | A3 | − |
|  | 400-VAMVFSQEDCM-410 (1) |  |  |
|  | VAMVFSQED | − | DR |
|  | 412-KAVRGDLNFVNRANQRLNPMHQLLRHFQKDAKVLF-446 (21) |  |  |
|  | KAVRGDLNF | B58, B62 | − |
|  | AVRGDLNFV | A2, A26 | − |
|  | VRGDLNFVN | − | DR |
|  | LNFVNRANQ | − | DR |
|  | NFVNRANQR | A3 | − |
|  | FVNRANQRL | A1, A2, B7, B8, B39, B62 | DR |
|  | VNRANQRLN | − | DR |
|  | NRANQRLNP | B27 | − |
|  | RANQRLNPM | A2, B7, B8, B58, B62 | − |
|  | QRLNPMHQL | B27, B39 | − |
|  | RLNPMHQLL | A1, A2, A3, A24, B8, B58, B62 | − |
|  | LNPMHQLLR | A2 | DR |
|  | PMHQLLRHF | A24, B62 | − |
|  | MHQLLRHFQ | − | DR |
|  | HQLLRHFQK | A3, B27 | − |
|  | QLLRHFQKD | A2 | − |
|  | LLRHFQKDA | A2 | − |
|  | LRHFQKDAK | B27 | DR |
|  | RHFQKDAKV | A2, B27 | − |
|  | HFQKDAKVL | A2, B8, B39 | − |
|  | FQKDAKVLF | A1, A24, B8, B27, B39, B44, B62 | DR |
|  | 479-RVSKMGVDEYS-489 (1) |  |  |
|  | VSKMGVDEY | A1, A26, B62 | − |
|  | 509-GNVLLSPEEVSETQG-523 (1) |  |  |
|  | NVLLSPEEV | A2 | − |
|  | 527-LTITYSSSMMWEINGPESVL-546 (6) |  |  |
|  | LTITYSSSM | A1, A2, A26, B7, B58, B62 | DR |
|  | TITYSSSMM | A26, B7 | − |
|  | ITYSSSMMW | A1, A3, A24, B58, B62 | DR |
|  | YSSSMMWEI | A1, A2, A24, B58 | DR |
|  | MMWEINGPE | B62 | − |
|  | WEINGPESV | B44 | DR |
|  | 548-NTYQWIIRNWE-558 (2) |  |  |
|  | NTYQWIIRN | A2, A3 | − |
|  | TYQWIIRNW | A24 | − |
|  | 570-MLYNKMEFEPFQSLVPKA-587 (5) |  |  |
|  | MLYNKMEFE | − | DR |
|  | YNKMEFEPF | B8, B62 | DR |
|  | MEFEPFQSL | A2, B39, B44, B62 | − |
|  | FEPFQSLVP | B44 | DR |
|  | EPFQSLVPK | A3 | − |
|  | 614-QIIKLLPFAAAPP-626 (4) |  |  |
|  | QIIKLLPFA | A2, A26 | − |
|  | IIKLLPFAA | B8 | DR |
|  | IKLLPFAAA | − | DR |
|  | LLPFAAAPP | − | DR |
|  | 628-QSRMQFSSLTVNVRGSGMRIL-648 (11) |  |  |
|  | QSRMQFSSL | A2, B7, B8 | − |
|  | SRMQFSSLT | B27 | − |
|  | RMQFSSLTV | A1, A2, A3, A24, B7, B8, B27, B62 | − |
|  | MQFSSLTVN | − | DR |
|  | QFSSLTVNV | A2 | − |
|  | FSSLTVNVR | − | DR |
|  | LTVNVRGSG | − | DR |
|  | TVNVRGSGM | A26, B7, B62 | − |
|  | VNVRGSGMR | − | DR |
|  | NVRGSGMRI | B7 | − |
|  | VRGSGMRIL | B27, B39 | DR |
|  | 685-GVESAVLRGFLI-696 (3) |  |  |
|  | VESAVLRGF | B44, B62 | − |
|  | ESAVLRGFL | A26 | − |
|  | SAVLRGFLI | A24, B7 | − |
| **PB1** | 1-MDVNPTLLFLKVP-13 (4) |  |  |
|  | MDVNPTLLF | A1, B44, B62 | DR |
|  | DVNPTLLFL | A2, A26 | − |
|  | VNPTLLFLK | A3 | − |
|  | NPTLLFLKV | B7, B8 | − |
|  | 15-QNAISTTFPYTGDPPYSHGTGTGYTMDTVNRTHQYSE-51 (13) |  |  |
|  | NAISTTFPY | A1, A3, A26, B8, B58, B62 | − |
|  | AISTTFPYT | A2 | − |
|  | ISTTFPYTG | − | DR |
|  | FPYTGDPPY | A1, A26, B7, B8, B62 | − |
|  | YTGDPPYSH | − | DR |
|  | YSHGTGTGY | A1, A3, A26, B27, B58, B62 | DR |
|  | HGTGTGYTM | B39 | − |
|  | GTGYTMDTV | A1 | − |
|  | GYTMDTVNR | A3 | − |
|  | YTMDTVNRT | A1, A2, A26 | DR |
|  | TMDTVNRTH | A1 | − |
|  | DTVNRTHQY | A1, A26, B62 | − |
|  | VNRTHQYSE | − | DR |
|  | 114-VQQTRVDKLTQGRQTYDWTLNRNQPAATALANTIE-148 (14) |  |  |
|  | VQQTRVDKL | A24, B62 | DR |
|  | RVDKLTQGR | A3 | − |
|  | KLTQGRQTY | A1, A2, A3, B8, B58, B62 | − |
|  | LTQGRQTYD | − | DR |
|  | TQGRQTYDW | B58 | − |
|  | GRQTYDWTL | B27, B39, B44 | − |
|  | QTYDWTLNR | A1, A2, A3 | − |
|  | WTLNRNQPA | − | DR |
|  | TLNRNQPAA | A2 | − |
|  | LNRNQPAAT | − | DR |
|  | NRNQPAATA | B27 | − |
|  | RNQPAATAL | B7, B8, B39, B44, B62 | − |
|  | NQPAATALA | A2 | − |
|  | AATALANTI | A2 | − |
|  | 196-TKKMVTQRTIGKKK-209 (4) |  |  |
|  | KKMVTQRTI | A24, B27 | − |
|  | MVTQRTIGK | A3 | DR |
|  | VTQRTIGKK | A3 | − |
|  | TQRTIGKKK | A3 | − |
|  | 337-LSIAPIMFSNKMARLGKGYMFESK-360 (12) |  |  |
|  | LSIAPIMFS | − | DR |
|  | SIAPIMFSN | A2, A3 | − |
|  | IAPIMFSNK | A3, A26 | − |
|  | APIMFSNKM | B7 | − |
|  | IMFSNKMAR | A3 | DR |
|  | MFSNKMARL | A24, B8 | DR |
|  | FSNKMARLG | − | DR |
|  | SNKMARLGK | A3 | − |
|  | KMARLGKGY | A1, A3, A26, B8, B27, B58, B62 | − |
|  | MARLGKGYM | B7, B8 | DR |
|  | ARLGKGYMF | A1, A24, B8, B27, B39 | − |
|  | RLGKGYMFE | A3 | − |
|  | 362-MKLRTQIPAEMLA-374 (4) |  |  |
|  | MKLRTQIPA | − | DR |
|  | KLRTQIPAE | A2 | − |
|  | LRTQIPAEM | B27 | DR |
|  | RTQIPAEML | A1, B58, B62 | − |
|  | 474-GINMSKKKSYIN-485 (3) |  |  |
|  | INMSKKKSY | B62 | DR |
|  | NMSKKKSYI | A2, B8 | − |
|  | MSKKKSYIN | − | DR |
|  | 487-TGTFEFTSFFYRYGFVANFSMELPSFGVSG-516 (17) |  |  |
|  | TGTFEFTSF | B58 | − |
|  | GTFEFTSFF | A1, A26, B58, B62 | − |
|  | TFEFTSFFY | A1, A3, A24, A26, B62 | − |
|  | FEFTSFFYR | A3, A2, B44 | DR |
|  | EFTSFFYRY | A1, A24, A26, B8 | − |
|  | FTSFFYRYG | − | DR |
|  | TSFFYRYGF | A26, B58, B62 | − |
|  | SFFYRYGFV | A24 | − |
|  | FFYRYGFVA | A2, B8, B39 | DR |
|  | FYRYGFVAN | − | DR |
|  | YRYGFVANF | A1, A26, B8, B27, B39, B62 | DR |
|  | RYGFVANFS | A24 | − |
|  | YGFVANFSM | B8, B39, B58, B62 | DR |
|  | FVANFSMEL | A1, A2, A3, A26, B7, B8, B39, B58, B62 | DR |
|  | ANFSMELPS | A3 | − |
|  | NFSMELPSF | A24 | − |
|  | SMELPSFGV | A1, A2 | − |
|  | 518-NESADMSIGVTVIKNNMINNDLGPATAQMALQLFIKDYR… |  |  |
|  | NESADMSIG | B44 | − |
|  | ESADMSIGV | A1, A2, A26 | − |
|  | ADMSIGVTV | A2, B44 | − |
|  | DMSIGVTVI | A2 | − |
|  | MSIGVTVIK | A3 | − |
|  | VTVIKNNMI | A1, A2, A24 | DR |
|  | TVIKNNMIN | A3 | − |
|  | IKNNMINND | − | DR |
|  | MINNDLGPA | A3 | DR |
|  | INNDLGPAT | − | DR |
|  | NNDLGPATA | A2 | − |
|  | LGPATAQMA | − | DR |
|  | GPATAQMAL | B7, B8, B39 | − |
|  | ATAQMALQL | A1,A2, A3, B7, B39, B58 | − |
|  | TAQMALQLF | A24, B58, B62 | − |
|  | AQMALQLFI | A2, A24, B44, B62 | − |
|  | QMALQLFIK | A2, A3 | − |
|  | ALQLFIKDY | A1, A2, A3, A26, B62 | − |
|  | LQLFIKDYR | − | DR |
|  | …QLFIKDYRYTYRCHRGDTQIQTRRSFE-575 (28) |  |  |
|  | QLFIKDYRY | A1, A3, A26, B8, B58, B62 | − |
|  | LFIKDYRYT | − | DR |
|  | FIKDYRYTY | A1, A3, A26, B8, B27, B62 | DR |
|  | IKDYRYTYR | − | DR |
|  | YRYTYRCHR | A1, A3, B27 | DR |
|  | YTYRCHRGD | − | DR |
|  | YRCHRGDTQ | − | DR |
|  | GDTQIQTRR | A3 | − |
|  | TQIQTRRSF | A24, B7, B27, B39, B62 | − |
|  | 656-MEYDAVATTHSW-666 (2) |  |  |
|  | MEYDAVATT | A2, B44 | DR |
|  | DAVATTHSW | B58 | − |
|  | 668-PKRNRSILNTSQRGILEDEQMYQ-690 (8) |  |  |
|  | KRNRSILNT | B27 | − |
|  | NRSILNTSQ | B27 | − |
|  | RSILNTSQR | A3 | − |
|  | ILNTSQRGI | A2 | DR |
|  | LNTSQRGIL | − | DR |
|  | RGILEDEQM | B58 | − |
|  | GILEDEQMY | A1, A26, B62 | − |
|  | ILEDEQMYQ | A2 | DR |
| **PA** | 29-KIETNKFAAICTHLEVCFMYSDFHFI-54 (12) |  |  |
|  | KIETNKFAA | A2 | − |
|  | IETNKFAAI | B8, B44 | − |
|  | KFAAICTHL | A2, A24, B44, B58 | − |
|  | FAAICTHLE | − | DR |
|  | AAICTHLEV | A2, A26 | − |
|  | CTHLEVCFM | A2, A26 | − |
|  | THLEVCFMY | A1, B39 | − |
|  | LEVCFMYSD | − | DR |
|  | EVCFMYSDF | A26 | − |
|  | VCFMYSDFH | A3 | DR |
|  | CFMYSDFHF | A24, B58 | − |
|  | FMYSDFHFI | A2, A24, B8, B39, B62 | DR |
|  | 130-YYLEKANKIKSE-141 (3) |  |  |
|  | YYLEKANKI | A2, A24, B39 | DR |
|  | YLEKANKIK | A1, A2, A3, A24 | − |
|  | LEKANKIKS | − | DR |
|  | 143-THIHIFSFTGEEMA-156 (2) |  |  |
|  | HIFSFTGEE | A3 | − |
|  | IFSFTGEEM | − | DR |
|  | 185-RGLWDSFRQSERGEETIEE-203 (5) |  |  |
|  | GLWDSFRQS | A2 | − |
|  | FRQSERGEE | − | − |
|  | RQSERGEET | B62 | − |
|  | QSERGEETI | A1 | − |
|  | SERGEETIE | B44 | − |
|  | 298-HEGEGIPLYDAIKC-311 (2) |  |  |
|  | HEGEGIPLY | A1, A26, B44, B62 | − |
|  | IPLYDAIKC | B7 | DR |
|  | 560-SRPMFLYVRTNGTSK-574 (6) |  |  |
|  | SRPMFLYVR | B27 | − |
|  | RPMFLYVRT | B7 | − |
|  | MFLYVRTNG | − | DR |
|  | FLYVRTNGT | A2, B8 | DR |
|  | LYVRTNGTS | − | DR |
|  | YVRTNGTSK | A3, B7 | DR |
| **NP** | 1-MASQGTKRSYEQMET-15 (3) |  |  |
|  | MASQGTKRS | A1 | − |
|  | ASQGTKRSY | A1, A26, B62 | − |
|  | GTKRSYEQM | A26, B8 | − |
|  | 35-GIGRFYIQMCTELKL-49 (5) |  |  |
|  | IGRFYIQMC | − | DR |
|  | GRFYIQMCT | B27 | − |
|  | FYIQMCTEL | A2, A24, B39, B44 | DR |
|  | YIQMCTELK | A3 | DR |
|  | IQMCTELKL | A2, A24, B27, B39, B44, B62 | DR |
|  | 66-MVLSAFDERRN-76 (2) |  |  |
|  | MVLSAFDER | A3, A26 | − |
|  | VLSAFDERR | A3 | − |
|  | 78-YLEEHPSAGKDPKKTGGPIY-97 (4) |  |  |
|  | LEEHPSAGK | A3 | − |
|  | HPSAGKDPK | B7 | − |
|  | PSAGKDPKK | A3 | − |
|  | DPKKTGGPI | B7, B8 | − |
|  | 110-LYDKEEIRRIWRQANNG-126 (4) |  |  |
|  | KEEIRRIWR | A3 | − |
|  | IRRIWRQAN | − | DR |
|  | RRIWRQANN | B27 | − |
|  | RIWRQANNG | A2 | − |
|  | 241-DQVRESRNPGNAEIEDL-257 (2) |  |  |
|  | QVRESRNPG | A3 | − |
|  | SRNPGNAEI | B27, B39 | − |
|  | 410-QPTFSVQRNLPF-421 (1) |  |  |
|  | FSVQRNLPF | A1, A26, B7, B8, B39, B58, B62 | DR |
| **M1** | 1-MSLLTEVETYVLSI-14 (4) |  |  |
|  | SLLTEVETY | A1, A2, A3, A26, B58, B62 | − |
|  | LLTEVETYV | A2 | − |
|  | LTEVETYVL | A1, B8, B39 | − |
|  | EVETYVLSI | A1, A2, A26 | − |
|  | 122-GALASCMGLIYNRMG-136 (4) |  |  |
|  | GALASCMGL | A2 | − |
|  | ALASCMGLI | A2, A3 | − |
|  | LASCMGLIY | A1, A3, A26, B58. B62 | DR |
|  | SCMGLIYNR | A3 | − |
|  | 175-HENRMVLASTTAKAMEQMAGSSEQAAEAME-204 (9) |  |  |
|  | NRMVLASTT | B27 | − |
|  | RMVLASTTA | A2, B62 | − |
|  | MVLASTTAK | A3 | DR |
|  | VLASTTAKA | A2, A3 | DR |
|  | LASTTAKAM | B7, B8, B62 | DR |
|  | TTAKAMEQM | A1, A26, B58 | − |
|  | EQMAGSSEQ | B62 | − |
|  | QMAGSSEQA | A2, B62 | − |
|  | SSEQAAEAM | A1 | − |
|  | 208-QARQMVQAMR-217 (2) |  |  |
|  | QARQMVQAM | B7, B8, B62 | − |
|  | ARQMVQAMR | A3, B27 | − |
